# Diagnostic Yield of Fluorescence and Ziehl-Neelsen Staining Techniques in the Diagnosis of Pulmonary Tuberculosis: A Comparative Study in a District Health Facility

**DOI:** 10.1155/2019/4091937

**Published:** 2019-04-10

**Authors:** Eben Godsway Dzodanu, Justice Afrifa, Desmond Omane Acheampong, Isaac Dadzie

**Affiliations:** ^1^Department of Medical Laboratory Science, University of Cape Coast, Cape Coast, Ghana; ^2^Scientific Research Center, The Second Affiliated Hospital of Harbin Medical University, Harbin, China; ^3^Department of Biomedical Sciences, University of Cape Coast, Cape Coast, Ghana

## Abstract

**Background:**

Despite the recent advancement in diagnostic methods, the smear microscopy remains the gold standard for the diagnosis of pulmonary tuberculosis in high burden countries like Ghana. Notwithstanding, fluorescence staining technique provides a more efficient option for the detection of* Mycobacterium tuberculosis* positive smears. This study therefore aimed at assessing the diagnostic performance of fluorescence microscopy (FM) and Ziehl-Neelsen (ZN) staining techniques in the diagnosis of pulmonary tuberculosis.

**Methods:**

A comparative study was carried out on 100 patients who reported at the Out Patients Department (OPD) or the Directly Observed Therapy (DOT) center of the Kade Government Hospital and were suspected of having pulmonary tuberculosis. Two (2) sputum samples each were collected. This included one spot and one morning sample. The smears were prepared and stained with FM and ZN staining techniques. Xpert MTB/RIF assay was also performed.

**Results:**

Of the 200 samples analyzed, 71 (35.5%), 46 (23.0%), and 84 (42.0%) were positive for pulmonary tuberculosis when FM, ZN, and XPERT MTB/RIF assays were used, respectively. The mean reading time of FM was three times faster than the ZN technique with very good acceptance (1.5min: 4.6min). The sensitivity and specificity of fluorescent staining to that of XPERT MTB/RIF assay were 84.5% and 100%, respectively, while those of ZN staining were 54.8% and 100%, respectively.

**Conclusion:**

For a routine laboratory test in a resource-limited setting, our study has demonstrated that fluorescence staining technique is a more sensitive test for the diagnosis of pulmonary tuberculosis as compared to the conventional ZN technique.

## 1. Introduction

Tuberculosis caused by* Mycobacterium tuberculosis *remains a major public health problem with approximately one-third of the world's population affected. In 2017, 10 million people were infected with tuberculosis and 1.6 million died from the disease. Over 95% of tuberculosis deaths occur in low- and middle-income countries.[1].

A faster, simpler, more accurate, and less expensive means of diagnosis of tuberculosis is necessary for the control of people infected with the disease as wells as preventing its spread in the community [2]. Various investigations can be used to help in the diagnosis of tuberculosis, and these include chest radiographs, clinical suspicion, staining for acid-fast bacilli, culture for mycobacteria, and nucleic acid amplification assays. Sputum smear microscopy is the most preferred and rapid test that is widely used for the detection and diagnosis of pulmonary tuberculosis [2, 3]. The bacilli in the sputum can be detected either by ZN or fluorescence staining techniques. Sputum microscopy is helpful to assess the response to treatment and to establish a cure or failure at the end of treatment.

In many developing countries, the diagnosis of tuberculosis is mostly based on the ZN staining technique [4]. The sensitivity of sputum smear microscopy by ZN method, however, is reported to be low and variable, ranging from 20% to 80%, often depending on the diligence with which specimens are collected, smears are made, and stained smears are examined [4–7]. This procedure leaves a significant number of cases undetected, especially if it becomes the only means of diagnosis. FM was introduced to improve the outcomes of sputum smear microscopy. The sensitivity of conventional FM provides far better yield and detection of positive smears than the ZN and takes less time to perform [8–10]. There is however a lingering doubt about the specificity of FM as there is the possibility of false positives which may be due to the incorporation of fluorochrome dyes by inorganic objects [11, 12]. Additionally, cost constraint is a limitation of the FM [13] especially, in a low to medium income countries such as Ghana.

In Ghana, diagnosis of pulmonary tuberculosis has involved the use of conventional light microscopy to examine ZN stained direct smears. The FM is gradually replacing ZN stained smear microscopy, while the use of molecular techniques for TB diagnosis is mainly limited to the teaching and regional hospitals. There is currently no documented evidence in the country evaluating the application of the techniques for the diagnosis of pulmonary tuberculosis. Hence, this study compares the diagnostic efficacy of these three methods for the detection of pulmonary diagnosis of tuberculosis.

## 2. Materials and Methods

This prospective study was carried out at Kade Government Hospital in the Eastern Region by trained biomedical scientists. Samples were collected between 08:00 am and 12:00 noon each day. The study population consisted of patients suspected of pulmonary tuberculosis and have reported at the OPD/DOT center of the Kade Government Hospital. A total of 100 patients were recruited for the study.

### 2.1. Sample Collection and Preparation

The study subjects (hundred patients) were requested to submit two sputum samples each in a clean, sterile, leak-proof, wide-mouth containers. In total, 200 samples were collected. One sample from each patient was taken on the spot and the subjects were provided with a second prelabeled container for a morning sample to be taken at home. Preparation of smear for staining was done as described elsewhere [14]. For each sample, the smears were made in duplicate. Positive and negative control smears were also prepared.

### 2.2. Ziehl-Neelsen (ZN) Staining Procedure

The smears were arranged in serial order on staining bridge, with smear side up and flooded with filtered 0.1% Carbol Fuchsin. The smears were steamed and allowed to stain for 5 minutes, rinsed with water, and drained. They were decolorized with 25% sulphuric acid for 5 minutes, rinsed with water, and drained. They were then counterstained with 0.1% methylene blue solution for 1 minute and rinsed with water. The smear was allowed to air dry and examined microscopically using the oil immersion (100x) objective.

### 2.3. Fluorescence Microscopy Staining Procedure

The smears were flooded with filtered 0.1% auramine for at least 20 minutes. They were then rinsed with water and drained. Acid alcohol decolorizing solution (0.5%) was applied on the smear for 30 to 60 seconds, rinsed with water, and drained. They were then flooded with 0.5% potassium permanganate counterstain for a maximum of 1 minute and rinsed with water. The smears were allowed to air dry and examined microscopically using the dry (40x) objective lens of an LED illumination-based fluorescence microscope (Zeiss primo star ilED).

### 2.4. Xpert MTB/RIF Assay Procedure

Xpert MTB/ RIF assay was performed following the protocol of the manufacturer (Cepheid Inc., Sunnyvale, CA, USA). Samples were collected in containers provided and treated with sample reagent in a proportion of 2:1 and incubated for 15 minutes at room temperature. Two milliliters (2 ml) of the reagent treated sample was pipetted into the sample chamber of the Xpert cartridge. The Xpert cartridge was then placed into the GeneXpert instrument system and run. Results were generated after 90 min.

### 2.5. Ethical Consideration

Study approval was sought from the authorities of the Kade Government Hospital. Written informed consent was sought from all participants before recruitment. Records were kept strictly confidential.

## 3. Data Analysis

Data were entered in Microsoft, and the statistical analysis was performed using SPSS software, version 23.0 (IBM Inc.). Data were expressed in percentages for the different variables. Kappa test was used to determine the correlation between diagnostic tests and the receiver operating characteristic curve was used to determine the sensitivity and specificity of tests. P-value <0.05 was considered statistically significant. Xpert MTB/RIF assay was taken as reference when ZN and fluorescence microscopy tests were compared. Samples that were positive and negative by GeneXpert were considered true positive and true negative.

## 4. Results

A total of 200 samples received from 100 individuals were screened for the presence of acid-fast bacilli (AFB) using the ZN and fluorescence microscopy staining techniques. In the absence of culture, Xpert MTB/RIF, which amplifies and detects specific gene target of* Mycobacterium tuberculosis, *was used as a reference.

Table 1 shows the results of the diagnostic tests used in the study. Of the 200 samples analyzed, 46 (23.0%), 71 (35.5%), and 84 (42.0%) were positive for pulmonary tuberculosis when ZN staining, fluorescence staining, and Xpert MTB/RIF assay were used, respectively. Of the 100 subjects recruited for the study, FM yielded 37 (37%) whereas ZN staining identified 25 (25%) of them to be infected with the AFB.

Table 2 compares the results of ZN and fluorescence staining with the Xpert MTB/RIF test. Of the 84 positive samples, 38 (45.2%) were missed by ZN staining technique and 13 (15.5%) were missed by FM. Both fluorescence and ZN staining techniques showed a positive correlation with Xpert MTB/RIF diagnostic technique; however, fluorescent staining had a stronger correlation compared to ZN staining (kappa=0.864, p≤0.001 versus kappa=0.584, p≤0.001). It could also be observed that all the samples that were negative by Xpert MTB/RIF test were also negative by both ZN and fluorescence staining.

**Figure 1:**
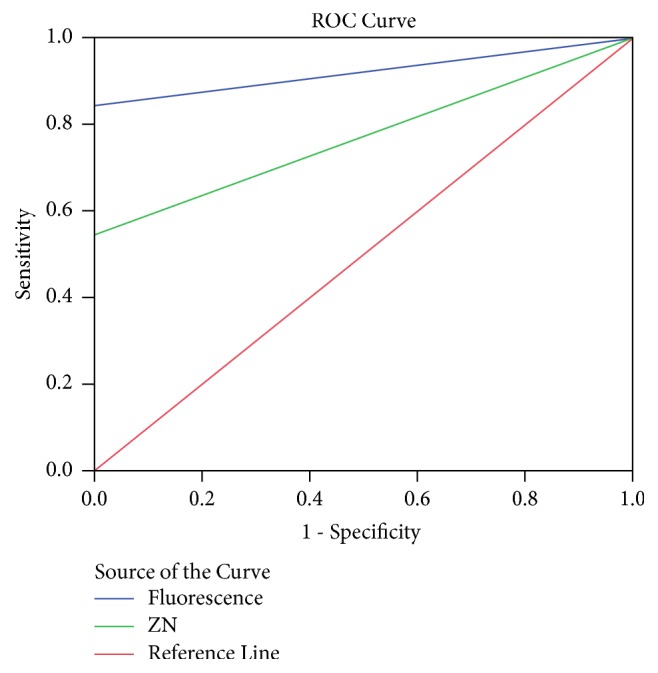
Receiver operator characteristic curve.

Tables 3 and 4 compare the diagnostic performance of ZN to that of FM. Fluorescence staining showed a positive correlation with ZN staining technique (kappa=0.680, p≤0.001). It could be seen that 25 (35.2%) of the samples that were positive by fluorescence microscopy were missed by ZN staining whereas only 1 (0.8%) sample that was positive by the ZN staining was negative by FM. The two tests had the same specificity (100%); however, the sensitivity of fluorescent staining (84.5%) was higher than that of ZN staining (54.8). The ROC analysis (Figure 1) also proved fluorescent staining (AUC= 0.923) to be a better diagnostic test than ZN staining (AUC= 0.774), when the tests were compared to the reference.

Table 5 shows the comparison of the diagnostic yield of spot and early morning sputum samples as stained by fluorescence and ZN staining techniques. Of the 37 early morning samples that tested positive for AFB, 34 (91.8%) of their corresponding spot samples also tested positive with 3(8.1%) testing negative when fluorescent staining technique was used. There was a strong agreement between early morning and spot samples as indicated by the Cohens Kappa (0.935); however, the difference was statistically significant (p<0.05). Also, of the 25 early morning samples that tested positive for AFB, 21 (84.0%) of their corresponding spot samples also tested positive with 4 (16.0%) testing negative when ZN staining technique was used.

**Figure 2:**
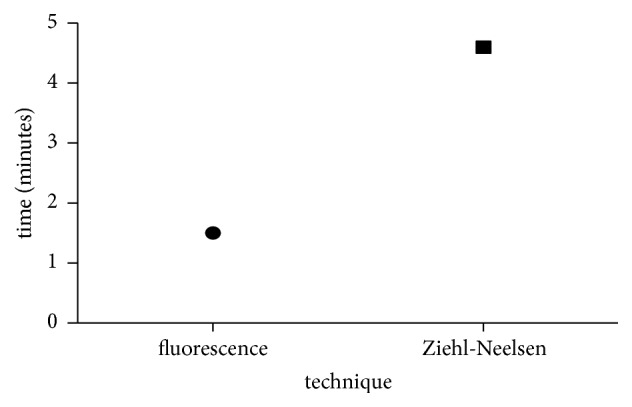
Average time taken to observe fluorescence and ZN stained smears.

The mean reading time of fluorescent staining technique was three times faster than the ZN technique with very good acceptance (1.5 min: 4.6 min) (Figure 2)

## 5. Discussion

Growth detection of AFB in culture considered the most sensitive method for diagnosis of TB is not routinely done in our health facilities mainly due to the slow growth of the bacteria and the lack of equipment required for the test. Prompt diagnosis of TB, therefore, is achieved by AFB smear microscopy, mostly by the ZN technique and recently by the fluorescence microscopy and the Xpert MTB/RIF in few facilities that have them. We compared the results of ZN stain smear and fluorescence staining with Xpert MTB/RIF test for detection of AFB in sputum samples.

Results of this study demonstrated that Xpert MTB/RIF diagnosed approximately 15% more of the 200 samples screened than FM and as high as 45% more than ZN. The superior performance of Xpert MTB/RIF over fluorescence and ZN microscopy in the diagnosis of pulmonary tuberculosis has been established in many studies [15–18]. Also, FM produced a higher diagnostic yield compared to that of ZN staining technique among our study samples. This finding confirms the previously reported superior performance of FM over the conventional ZN technique for AFB detection [19–21]. Comparatively, the fluorescence microscopy generated readings at a rate that is approximately three times faster than ZN technique, thus helping save approximately 2 minutes per slide (66%) which corroborate findings of a study conducted by Marais, Brittle [22]. Therefore, the introduction of LED-FM would be time-saving and allow for quality microscopy.

The study revealed a higher percentage of false negatives from ZN staining technique (45.2%) as compared to FM (15.5%) and this is consistent with a study carried out in Sinamangal, Nepal [19], which showed that the percentage of false negative by FM staining was only 2.78%, and was in sharp contrast to that of ZN (40.27%). FM was also able to detect more paucibacillary cases than ZN. The better case detection rates of FM over ZN were comparable to reports found in several studies [8, 23–25].

A significant benchmark for an alternative diagnostic method is its ability to establish a linear relationship with the gold standard. Interestingly, both FM and ZN techniques correlated positively with Xpert MTB/RIF diagnostic technique. Fluorescent staining, however, showed a stronger linear relationship as compared to ZN staining (kappa=0.864, p≤0.001 versus kappa=0584, p≤0.001). Elsewhere, Stella et al. observed similar findings when both the FM and ZN techniques were compared to the PCR method (kappa FM versus PCR=0.60; ZN versus PCR=0.54) [26].

In an ideal situation, a diagnostic technique should be 100% specific and 100% sensitive. Our results indicated that both the ZN and FM techniques showed similar specificity (100%) when compared to that of Xpert MTB/RIF; however, in relation to Xpert MTB/RIF, FM (84.5%) was more sensitive than ZN staining (54.8%). In general, it has been reported that, in the diagnosis of pulmonary tuberculosis, the fluorescence staining technique provides better sensitivity and specificity when compared to the ZN [27, 28].

This study showed that, in both FM and ZN staining techniques, early morning samples yielded more AFB as compared to spot samples. Interestingly, sputum samples positive for smear collected at the spot were also positive for samples collected early in the morning. This agrees with a study carried out by Myneedu et al. and which showed that the first sputum sample (spot sample) collected immediately in the vicinity of the laboratory showed a reduced smear positivity as compared to morning samples. Similarly, they also reported that all AFB positive spot samples were also positive for AFB in the early morning smears [29]. To reduce diagnostic defaulting, this result calls for a second look at the standard 2-day protocol of collecting samples on two consecutive days for diagnosis of pulmonary tuberculosis. Early morning samples prove to be ideal when onetime sample collection is to be adopted.

In conclusion, our findings show the FM technique to be of a more diagnostic value compared with the ZN technique. It is more sensitive and can detect accurately paucibacillary cases and this has implication on early treatment of pulmonary tuberculosis.

## Figures and Tables

**Table 1:** Comparison of diagnostic tests used.

	Fluorescent staining			Ziehl-Neelsen staining		Xpert MTB/Rif	
	Spot	EM	(N=200)	Spot	EM	(N=200)	(N=200)
	(N=100)	(N=100)		(N=100)	(N=100)		
Positive	34(34)	37(37)	71 (35.5)	21(21)	25(25)	46 (23.0)	84 (42.0)
Negative	66(66)	63(63)	129 (64.5)	79(79)	75(75)	154 (77.0)	116 (58.0)
			*Sputum Grading*				
Scanty			12 (6.0)		2 (1.0)		
1+			20 (10.0)		10 (5.0)		
2+			21 (10.5)		24 (12.0)		
3+			18 (9.0)		10 (5.0)		

Data is represented in raw figures and percentages. EM: early morning sample.

**Table 2:** Correlation of fluorescence and ZN staining techniques with Xpert MTB/Rif.

Staining Technique	XPERT MTB/Rif		Kappa	P-value	PPV (%)	NPV (%)
	Positive	Negative				
*Fluorescence*						
Positive	71 (84.5)	0 (0.0)	0.864	p≤0.001	100	92.1
Negative	13 (15.5)	116 (100)				
*ZN*						
Positive	46 (54.8)	0 (0.0)	0.584	p≤0.001	100	77.3
Negative	38 (45.2)	116 (100)				

Data is represented in raw figures and percentages. P-value is statistically significant if p < 0.05 as compared between Xpert MTB/Rif and fluorescence microscopy, Xpert MTB/Rif and ZN.

**Table 3:** Comparison of diagnostic performance of fluorescence staining to ZN staining.

ZN	Fluorescence		Kappa	P-value
	Positive	Negative		
Positive	46 (64.8)	1 (0.8)	0.680	p≤0.001
Negative	25 (35.2)	128 (99.2)		

Data is represented in raw figures and percentages. P-value is statistically significant if p < 0.05 as compared between FM and ZN.

**Table 4:** Receiver operator characteristic (ROC) curve parameters.

Technique	Sensitivity	Specificity	AUC	95%CI	P-value
Fluorescence	84.5%	100%	0.923	0.876-0.969	p≤0.001
ZN	54.8%	100%	0.774	0.702-0.846	p≤0.001

*Xpert MTB/Rif used as reference diagnostic test; AUC: area under curve. *Data is represented in percentages. P-value is statistically significant if p < 0.05 as compared between Xpert MTB/Rif and fluorescence microscopy, Xpert MTB/Rif, and Ziehl-Neelsen.

**Table 5:** Comparison of diagnostic yield of “spot” and “early morning” sputum samples.

Spot	EM		Kappa	P value
	Positive	Negative		
*Fluorescence*				
Positive	34(91.8)	0(0.0)	0.935	p≤0.001
Negative	3(8.1)	63(100.0)		

*ZN*				
Positive	21(84.0)	0(0.0)	0.887	p≤0.001
Negative	4(16.0)	75(100.0)		

Data is represented in raw figures and percentages. P-value is statistically significant if p< 0.05 as compared between “spot” and “early morning (EM)” sputum samples. EM: early morning.

## Data Availability

The data used to support the findings of this study are available from the corresponding author upon request.
